# On Temperature

**Published:** 1804-10-01

**Authors:** 


### *To the Editors of the Medical and Physical Journal.*

GENTLEMEN,

YOUR Correspondent, Dr. Blegborough, discovers a *cocoëthes scribendi*, that almost necessarily betrays him into a *cacoëthes errandi*. That multifarious writer conceives his notions to be so many *fiats*, to which implicit acquiescence should be given. His speculative wanderings, how-

 ever, ever, are not scientific truths; nor do they tend to illustrate the practical difficulties he would wish to explain.

The Doctor's view of temperature, in your last Number, presents a singular example of inconsistent inquiry. He imagines heat to pervade and fill the animal body, as water may occupy a sponge; and as that fluid may be pressed from the latter, so the matter of heat, the Doctor thinks, may be abstracted from the former. Were this the case, the most refrigerant process that could be instituted would be brisk friction; and, consequently, instead of cold water being applied as an appropriate remedy to an inflamed part, mechanical rubbing would more readily squeeze out the morbid excess of heat.

The Doctor speaks at one time of heat being a stimulant, sufficiently powerful to excite the worst forms of typhus fever; and at another affirms, that it so expands the vessels, as to render them incapable of contractile exertion. But, notwithstanding this vascular inaction from excessive heat, the Doctor acknowledges that the various phenomena of violent fever still obtain.

The Doctor also, with licentious speculation, aspires to inform the public, and particularly that distinguished medical philosopher, Dr. Currie, that the exacerbation and remission of febrile heat are governed by the different degrees of atmospheric temperature, at the periods when these changes respectively occur. The Doctor then as familiarly speaks of removing a pint measure of material heat as he would of an equal quantity of blood, and deduces important mechanical relief to the system, and particularly to the brain, from the abstraction of that expansive fluid.

This very visionary reasoning has been induced by a total forgetfulness that the subject of inquiry related to animal life, endowed with the power of generating salutary heat, agreeably to the laws of repulsive motion, and of transferring to surrounding media that which may be redundant; that if the atmospheric temperature be freely applied, no morbid surplus of heat is likely to occur; and that when it does present, it in no shape mechanically and stationarily amasses, but that it directly results from the morbid action of vessels generating the exuberant portion of that motive principle.

The mere abstraction of the Doctor's morbid accumulation of heat, can have but little influence in curatively reducing either the excessive temperature of typhus fever, or that of inflammatory affection; the abstraction must be uninterruptedly uninterruptedly continued, until the diseased action which furnishes it be superseded by that which is healthful, and which is recognised by the sense of salutary heat.

Instead of vital heat being an extraneous something, mechanically running through the animal system, and either generally or partially amassing and proceeding to febrile or inflammatory excess, it is the very principle of living motion, actuating, regulating, and equipoising the various functions of the animal economy; its offensive excess is obviated by the evaporating or abstracting agency of cuticular perspiration, and that of the various other secretions.

When obstacles occur to these refrigerating outlets, redundant heat will arise, which may variously disorder the motive conditions of healthful excitability; sympathies may be also engendered, which may diversify the external character of the affection. Under these circumstances, the indication of immoderate heat afforded as well by the natural sense of feeling as by thermometrical test, will direct to the suitable reduction of temperature. The mode of applying this relief may be adapted to the temperamental sensibility of patients; but it will in general be found, that Dr. Currie's plan of suddenly dashing cold water over the surface of the skin, will at once effectually dissolve the morbidly associated actions which may have been formed, and promptly transfer the redundant heat. The mode by sponging may also ultimately avail; but it is much less likely to produce the salutary change in the inordinately generative action of heat, and its diseased sympathies, than the more impressive shock induced by its instantaneous application.

Dr. B. is on philosophic ground, in investigating the influence of animal temperature on diseases; but his inquiry must be neither gratuitously nor incongruously conducted. The Doctor must not advance as tenable, doctrine, which, to unprejudiced reason, appears indefensible, without the aid of facts to justify its recommendation; nor must he, at one time, contend that morbid excess of temperature ought to be reduced by *cold water*, and that, at another, *steaming heat* or the *vapour bath*, is best adapted to produce that effect!

Less disposition to assert, and a more earnest endeavour to correct and mature, crude and hasty views of the motive laws of the animal economy, may qualify the Doctor for intricate researches in Medical Philosophy, and afford him a better title than he has yet discovered, for offering hints of practical improvement in the theory and management of diseases of temperature, to so able an investigator as the much and deservedly respected *Author of Reports* on that subject.

CANDIDUS.

*St. James's Street, Sept.* 12, 1804.

